# The quality of life of parents of children with disabilities in Saudi Arabia: a systematic review

**DOI:** 10.3389/fped.2026.1660247

**Published:** 2026-03-06

**Authors:** Abdullah Ahmed Alghamdi, Mohammad S. Alzahrani, Atiah H. Almalki, Majed A. Algarni

**Affiliations:** 1 Department of Special Education College of Education, Taif University, Taif, Saudi Arabia; 2 Department of Clinical Pharmacy, College of Pharmacy, Taif University, Taif, Saudi Arabia; 3 Department of Pharmaceutical Chemistry, College of Pharmacy, Taif University, Taif, Saudi Arabia; 4 Addiction and Neuroscience Research Unit, Health Science Campus, Taif University, Taif, Saudi Arabia

**Keywords:** attention-deficit/hyperactivity disorder (ADHD), autism spectrum disorder (ASD), cerebral palsy (CP), children with disabilities, down syndrome (DS), quality of life (QoL)

## Abstract

**Introduction:**

The quality of life (QoL) of parents and caregivers of children with disabilities in Saudi Arabia is examined in this systematic review.

**Methods:**

Fourteen cross-sectional studies published between 2020 and 2024 are included, encompassing 1,841 caregivers, of whom 60.2% are mothers, 23.1% fathers, and 18.4% other caregivers, caring for 1,460 children with disabilities. QoL is primarily assessed using the WHOQOL-BREF in 10 studies, followed by the SF-36 in two studies and the Beach Center Family Quality of Life Scale in one study.

**Results:**

Autism spectrum disorder (ASD), attention-deficit/hyperactivity disorder (ADHD), cerebral palsy (CP), and Down syndrome (DS) are the most commonly reported conditions. Negative QoL outcomes are reported in at least one domain in 9 of the 10 WHOQOL-BREF studies. The physical domain is most frequently affected (50% of studies), followed by the social and environmental domains (40% each) and the psychological domain (30%). More than half of caregivers (53.6%) are reported to be unemployed, and poorer QoL is consistently associated with unemployment, lower income, limited education, and restricted access to support services.

**Conclusion:**

Lower QoL is most frequently reported by mothers and by caregivers of children with severe or multiple disabilities, highlighting the need for targeted support interventions.

## 1 Introduction

**Figure 1:**
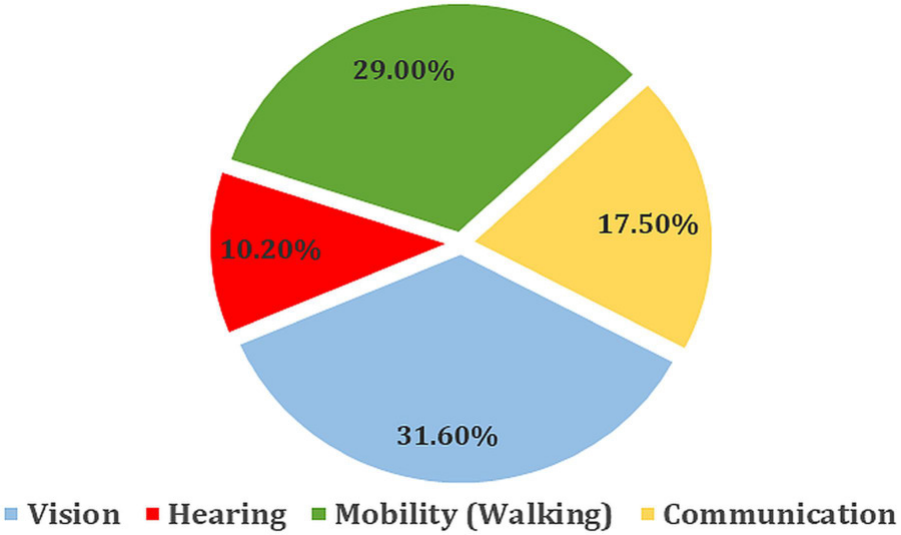
Percentage distribution of disabilities in children aged 2–4 years by type and severity (5).

It has been shown that understanding disabilities among children is critical for public health surveillance, child welfare promotion, and the development of an inclusive society, which also provides a framework for analyzing how the needs of vulnerable populations evolve in relation to social, health, and environmental factors (1, 2). In Saudi Arabia, a nation that blends traditional and modern values, there has been a growing focus on examining disabilities in children under 18, as the country's unique cultural and social context is leading towards the need for more context-sensitive evidence (3, 4). In 2022, it was shown that a national survey in Saudi Arabia revealed that 1.8% of the population had disabilities, which can be employed to understand the prevalence and distribution of disability in the population. It was shown that 41.5% of children experienced a single disability, while 58.5% had multiple disabilities, which may increase the demand for integrated services. Vision impairments (31.6%), mobility issues (29%), communication difficulties (17.5%), and hearing impairments (10.2%) (see Figure 1) are the most prevalent, which could thus be used to prioritize healthcare and social support initiatives. The primary causes included illnesses (39.2%), congenital factors (26.9%), aging (16.1%), and traffic accidents (4.7%), which could thus be used to inform prevention strategies and interventions based on more than aggregate population statistics (5).

Age, gender, socioeconomic status, and family structure are factors that are associated with how children and their families handle disabilities, thereby influencing both coping mechanisms and access to resources (6). Despite progress in addressing childhood disabilities in Saudi Arabia, challenges like unequal service access remain (7). This study is the first to explore the prevalence of disabilities among Saudi children, their coping strategies, and how social and personal factors influence their QoL, providing insights for evidence-based policies and targeted support (7–9).

Although caregiver and parental QoL have been examined internationally (10–15), the Saudi evidence base remains uneven and difficult to compare (6, 8, 14, 16). Most Saudi studies focus on one disability group at a time [e.g., autism or cerebral palsy (CP)], use cross-sectional designs, and rely on samples drawn from single settings or regions, which limits generalizability and prevents a clear national picture of parental QoL (1, 4, 8). In addition, findings are reported using different QoL instruments (e.g., WHOQOL-BREF, SF-36, Beach Center Family QoL) (13, 14), creating methodological heterogeneity that makes it hard to synthesize results consistently across conditions and caregiver groups. A Saudi-specific systematic review has been published (2023) (3), but the field has continued to develop, with newer studies (including 2024, 2025) extending evidence on family QoL and caregiver outcomes (6, 8, 14, 16). Furthermore, research guidance shows that using systematic methods, registered protocols, and careful quality checks helps reduce bias and produce more reliable conclusions (17–19). Therefore, an updated synthesis is needed to (i) integrate the expanding Saudi evidence across disability categories (1–4, 6, 8), (ii) summarize the most consistent determinants of poorer parental QoL (e.g., socioeconomic disadvantage and caregiver gender), and (iii) interpret these findings within Saudi Arabia's service and policy environment, where access to rehabilitation and family support is a key practical issue (5, 8). Accordingly, this systematic review updates and consolidates Saudi evidence on the QoL of parents/caregivers of children with disabilities and identifies the most consistent factors associated with poorer outcomes to inform targeted support and service planning.

### 1.1 Objectives and research questions

This systematic review seeks to evaluate the QoL of parents of children with disabilities in Saudi Arabia, which is especially important as it may be used to inform evidence-based interventions and policy enhancements. The objectives of this systematic review are structured around the research questions outlined in Table 1.

**Table 1:** Objectives and research questions.

No.	Research question	Primary focus/description
1	How does the quality of life of parents or caregivers vary across different sociodemographic factors?	Examines the influence of gender, employment status, education level, marital status, and income on caregiver quality of life to identify disparities and vulnerable groups.
2	How do different types and severities of disabilities affect the quality of life of parents?	Assesses the impact of autism spectrum disorder (ASD), attention deficit hyperactivity disorder (ADHD), cerebral palsy (CP), and Down syndrome (DS), as well as the relationship between disability severity and caregiver burden.
3	Are there differences in QoL outcomes between mothers and other caregivers such as fathers or extended family members?	Compares quality of life outcomes across caregiver roles to identify gender related caregiving burdens and differences in coping mechanisms.
4	Which quality of life domains are most affected including physical, psychological, social, and environmental domains?	Identifies the domains of caregivers' lives that are most impacted to inform targeted support and resource allocation.
5	How do social and governmental support systems affect the quality of life of parents of children with disabilities?	Evaluates the role of rehabilitation services, financial support, and societal interventions in improving caregiver well-being and reducing caregiving burden.

## 2 Literature review

There has been a growing recognition that ASD is a chronic neurodevelopmental condition, which can be used to explain difficulties in social interaction, communication, and repetitive behaviors or restricted interests. In Saudi Arabia, it was shown that approximately 1 in 40 children aged 2–4 years is diagnosed with ASD (20, 21). ASD varies in severity, with differing requirements for communication, social skills, and behavioral support, which is leading towards more context-specific interventions. Many children with ASD also have co-existing challenges, such as cognitive delays, language difficulties, and anxiety, which could be used to understand the daily functional limitations and support needs of these children (22).

Alnahdi et al. (23) conducted a study that looked at family quality of life among caregivers in Saudi Arabia, which examined those caring for individuals with ASD, intellectual and other disabilities, as well as those without disabilities (23). Using the Arabic version of the Beach Center Family Quality of Life Scale, the study assessed domains including family interaction (FI), parenting practices (PP), emotional well-being (EW), physical and material well-being (PMW), and disability-related support (DRS), which could be employed to identify areas where support is most required. Significantly higher satisfaction was reported by caregivers of individuals without disabilities, particularly in emotional well-being (EW) and physical and material well-being (PMW) (23). Lower satisfaction was generally reported by fathers compared to mothers, especially in family interaction (FI) and parenting practices. EW was the most affected domain, highlighting the need for improved emotional support. Caregiving for individuals with ASD remains particularly challenging, and tailored societal and policy support is essential to reduce caregiver burden (23) (see Table 2).

**Table 2:** Summary of studies on QOL in parents and children with disabilities.

References	Study objective	QOL measures	Disease/condition	Findings/results
Alnahdi et al. (23)	Family QOL of caregivers in Saudi Arabia	Beach Center Family Quality of Life Scale	ASD, intellectual disabilities, and others.	Caregivers of individuals with ASD reported lower satisfaction, especially in emotional and material well-being. Fathers reported lower satisfaction with family interaction and parenting. Gaps in emotional and material support were highlighted.
Al-Janabi et al. (24)	To identify independent demographic and social predictors of parents' quality of life so support can be directed to those most in need.	QOL and social support were measured using a structured self-administered questionnaire assessing multiple domains of QoL (e.g., health perception, physical functioning, mental and social functioning).	ASD	Parents experienced lower QoL, especially in mental and social domains. Factors associated with poorer QoL included being a mother, having a chronic condition, having a female child or a child with comorbidities. Marital satisfaction was linked to better QoL in most areas. Multiple regression was significant for all domains except role functioning (*p* = 0.258).
Manee et al. (29)	QOL of Arab mothers caring for children with disabilities in Kuwait	WHOQOL-BREF	Autism, CP, DS, developmental delay	Mothers of children with disabilities had significantly lower QOL across all domains compared to those with typical children. The nuclear family was the primary support source, while formal support systems were largely unavailable.
Romaniuk et al. (31)	Family QOL for children with ASC, ADHC, or both conditions	Family QOL Scale	Autism, ADHD	Families of children with both conditions reported lower emotional well-being than those with ASC alone. Highlighted the need for further research into dual-condition challenges.
Alhammad et al. (32)	Impact of walking ability on QOL in children with DS in Saudi Arabia	Pediatric QOL Inventory	DS	Children with DS walked shorter distances and had lower QOL in physical, social, and school domains. Early interventions to improve walking ability were recommended.
Asdaq et al. (45)	Relationship between depression, QOL, and COVID-19 challenges	WHOQOL-BREF	Disabilities during COVID-19	Depression higher in unvaccinated individuals. Vaccination and physical activity linked to better QOL. Most participants reported satisfactory overall QOL but low satisfaction in physical and psychological health.
Asiri et al. (37)	To summarize the overall QoL of caregivers of children with disabilities in Saudi Arabia and identify factors affecting caregivers' well-being.	Most studies used WHOQOL-BREF, SF-36, or similar tools to measure caregivers' physical, psychological, social, and environmental QoL	Children with various disabilities (e.g., CP, ASD, DS, learning disabilities) cared for by family caregivers in Saudi Arabia.	Caregivers of disabled children had reduced QoL in multiple domains, especially physical, psychological, and social relationships. Factors such as gender, age, and low income were found to negatively impact caregiver QoL.

The study by Al-Janabi et al. (24) examined the QoL of 248 parents of children with ASD to identify key factors affecting their well-being. The results showed that parents experience lower QoL due to psychological, financial, and social challenges. Mothers, parents with chronic conditions, and parents of female children or children with comorbidities reported significantly lower scores in health perception, mental health, and social functioning. Marital satisfaction was positively associated with most QoL domains. The authors suggest, and this is supported by several other studies, that these high-risk parent groups should be prioritized for support and interventions to improve family functioning and social support (24–27).

Emam et al. (28) studied parents of children with disabilities in Oman, Saudi Arabia, and Qatar and found that parenting stress was higher in Oman and Qatar. Parents with less support from family and friends reported lower life satisfaction. Social support was shown to buffer stress caused by parental distress, difficult child behavior, and poor parent-child interactions, thereby improving overall well-being. Fahad Manee et al. (29) compared the QoL of mothers in Kuwait who cared for children with disabilities (ASD, CP, DS, or developmental delay) with mothers of children without disabilities. Using the Arabic WHOQOL-BREF (30), it was shown that mothers of children with disabilities had significantly lower scores in all domains: physical (11.4 vs. 14.5), psychological (11.6 vs. 14.6), social (12.8 vs. 16.4), and environmental (12.4 vs. 14.8), with *p* < 0.001. These mothers relied mainly on their nuclear families for support because formal services were limited. It is noteworthy that occupational therapists play a key role in improving caregiver integration and access to services, which can enhance family well-being (29).

Romaniuk et al. (31) reported comparable overall family quality of life among mothers of children with ASD, ADHD, or both; however, emotional well-being was lower in mothers of children with ASD and ADHD comorbidity, indicating greater caregiving challenges. Alhammad et al. (32) found that Saudi children with Down syndrome had reduced physical, social, and school quality of life linked to impaired walking ability, despite relatively higher emotional well-being, underscoring the value of early mobility interventions (see Table 2). Additionally, migraine was shown to substantially impair daily functioning and emotional quality of life among women in Saudi Arabia, with over half experiencing severe disability, highlighting the need for improved clinical management of both disability and quality of life outcomes (33–36).

The study by Asiri et al. (37) conducted a review to examine the QoL of caregivers of children with disabilities in Saudi Arabia. The authors performed a comprehensive search of electronic databases and gray literature, which initially identified 233 studies. After applying inclusion criteria, 12 studies specifically focused on the QoL of caregivers of children with disabilities in the Saudi context. The review found that caregivers of children with disabilities have lower QoL, especially in physical, psychological, and social areas, with gender, age, and low income increasing their vulnerability. Additionally, a study comparing movement and QoL in 67 children (29 with DS, 38 typically developing) found no major differences in activity levels, though typically developing children had higher overall QoL (38). In children with DS, moderate physical activity was modestly linked to better school functioning, thereby suggesting that promoting movement can be used to improve overall outcomes.

It is evident from these studies that parental and caregiver QoL is influenced by multiple factors, including the type and severity of the child's disability, comorbid conditions, socioeconomic status, coping strategies, social and family support, mobility, and other health or environmental factors (14, 16, 18, 23, 26–28, 31, 39). EW, physical health, social participation, and access to support services were consistently identified as the most affected domains. It has been shown that adaptive coping, strong social support, early intervention programs, and policy measures to improve family resources are essential to enhance the QoL of parents and caregivers of children with disabilities in Saudi Arabia (1, 2, 8, 14, 23). These findings provide the foundation for effective planning of interventions and policies, which can alleviate multidimensional burdens and promote inclusive, supportive care systems (1, 2, 8, 14, 23).

## 3 Methodology

### 3.1 Inclusion and exclusion criteria

This review included quantitative studies that met specific inclusion and exclusion criteria. Studies were included if they focused on the QoL of parents or primary caregivers of children with disabilities in Saudi Arabia. Various disability conditions were considered, including attention-deficit/hyperactivity disorder (ADHD), cerebral palsy (CP), autism spectrum disorder (ASD), physical and sensory disabilities, intellectual and developmental disabilities, and Down syndrome (DS).

The age range of parents included in the studies was 23 years to over 57 years, while caregivers ranged from 14 to 57 years. Children included in the studies ranged in age from infancy to 18 years. Only studies that assessed QoL using validated instruments were included. These instruments comprised the WHOQOL-BREF (including its Arabic version), PedsQL 3.0 CP Module, the Beach Center Family QoL Scale, SF-36, and RAND SF-36.

Eligible studies were required to compare parental QoL across different demographic and child-related factors. Such factors included parent gender, employment status, education, housing, and type of disability. Comparisons were also made between mothers and fathers, caregivers of children with CP, ADHD, or ASD, and families with children with or without disabilities. In addition, studies examining income level, coping strategies, marital status, and experiences of discrimination were considered.

Exclusion criteria were applied to ensure the reliability of findings. Studies involving children with comorbid chronic illnesses were excluded, as these conditions could confound QoL outcomes. Intervention-based studies that evaluated the effects of treatments or programs on parental QoL were also excluded. Furthermore, only studies published in English between January 2020 and December 2024 were included to maintain consistency in language and time frame.

### 3.2 Search procedures

An initial search of the Cochrane Database of Systematic Reviews and PROSPERO was conducted in order to identify any existing or registered systematic reviews on the QoL of parents of children with disabilities in Saudi Arabia (17). Following this, a comprehensive literature search was carried out across multiple electronic databases, including PubMed, Scopus, Embase, Web of Science, CINAHL, ERIC, the Cochrane Library, Google Scholar, the Saudi Digital Library, and NARIC. All databases were searched using the same time limits and core eligibility filters, restricting results to studies published between January 2020 and December 2024, written in English, and involving human participants. This systematic approach was applied in order to understand the most recent evidence and to be able to capture all relevant studies within a defined time frame. It has been shown that using multiple databases, as well as being thorough in search methods, increases the likelihood that all eligible studies are identified (18).

A structured search strategy was developed, combining controlled vocabulary such as MeSH terms where applicable and free-text keywords. Boolean operators “AND” and “OR” were applied to combine search terms. The search string focused on parental and caregiver populations, different types of disabilities including autism spectrum disorder, ADHD, cerebral palsy (CP), Down syndrome (DS), and other physical disabilities, QoL outcomes, and Saudi-specific studies. Database-specific filters were applied in order to improve precision. In PubMed, filters for publication date, English language, human participants, and study type (observational and cross-sectional studies) were applied. In Scopus and Web of Science, searches were limited to peer-reviewed journal articles within health, social science, and psychology subject areas published between 2020 and 2024. Embase searches were restricted to human studies, English language, and observational study designs. CINAHL and ERIC were filtered for caregiver or parent populations, quality-of-life outcomes, English language, and the specified publication period. The Cochrane Library was searched in order to confirm the absence of prior Saudi-specific syntheses. Google Scholar was used as a supplementary source, and the first 200 results sorted by relevance were screened. In addition, the Saudi Digital Library and NARIC were searched for peer-reviewed articles matching the same keywords and publication period.

During the screening stage, additional inclusion and exclusion criteria were applied based on parental employment status (full-time, part-time, unemployed), parental and child age, and type of disability, in line with the predefined eligibility criteria. Studies examining the caregiving role and its association with parental QoL across disability types were included to inform caregiver well-being, policy, and practice. Most eligible studies were published between 2020 and 2024, with publications grouped into early (2020–2021), mid (2022–2023), and recent (2024) periods, demonstrating a clear upward trend in research activity. Reference lists were also manually screened to ensure comprehensive study identification.

### 3.3 Methodological quality assessment

The methodological quality of the reviewed studies was carefully assessed to ensure that the review findings are reliable and valid. A systematic evaluation process was employed to enhance transparency and maintain methodological rigor. The PRISMA 2020 guidelines (Page et al. 40) informed the assessment, as they provide a clear outline of how to report and assess the possibility of bias, supporting a consistent and reproducible review process. It is the method, which considers the quality of every study and its adherence to general methodological principles, that is used due to the necessity of reliable evidence (40).

The quality of the studies was checked with the help of predefined criteria, which is a systematic process that is currently being employed in order to make the review transparent and credible. These criteria were designed to include study design and methods, sampling strategy and sample size, validity and reliability of QoL measurement tools, appropriate application of data analysis approaches, consideration of potential confounding factors, ethical approval, clear reporting of results, and relevance to parental QoL in Saudi Arabia, allowing for a consistent assessment (19). Studies were rated as Good (high quality), Moderate (with some limitations), or Low (with major weaknesses). To reduce assessment bias, all included studies were evaluated independently by two reviewers, a process aimed at ensuring consistency and reliability. Cohen's Kappa was used to measure agreement between the reviewers, as it helps show how consistent the study quality ratings were beyond chance.

## 4 Results

### 4.1 Study selection

**Figure 2:**
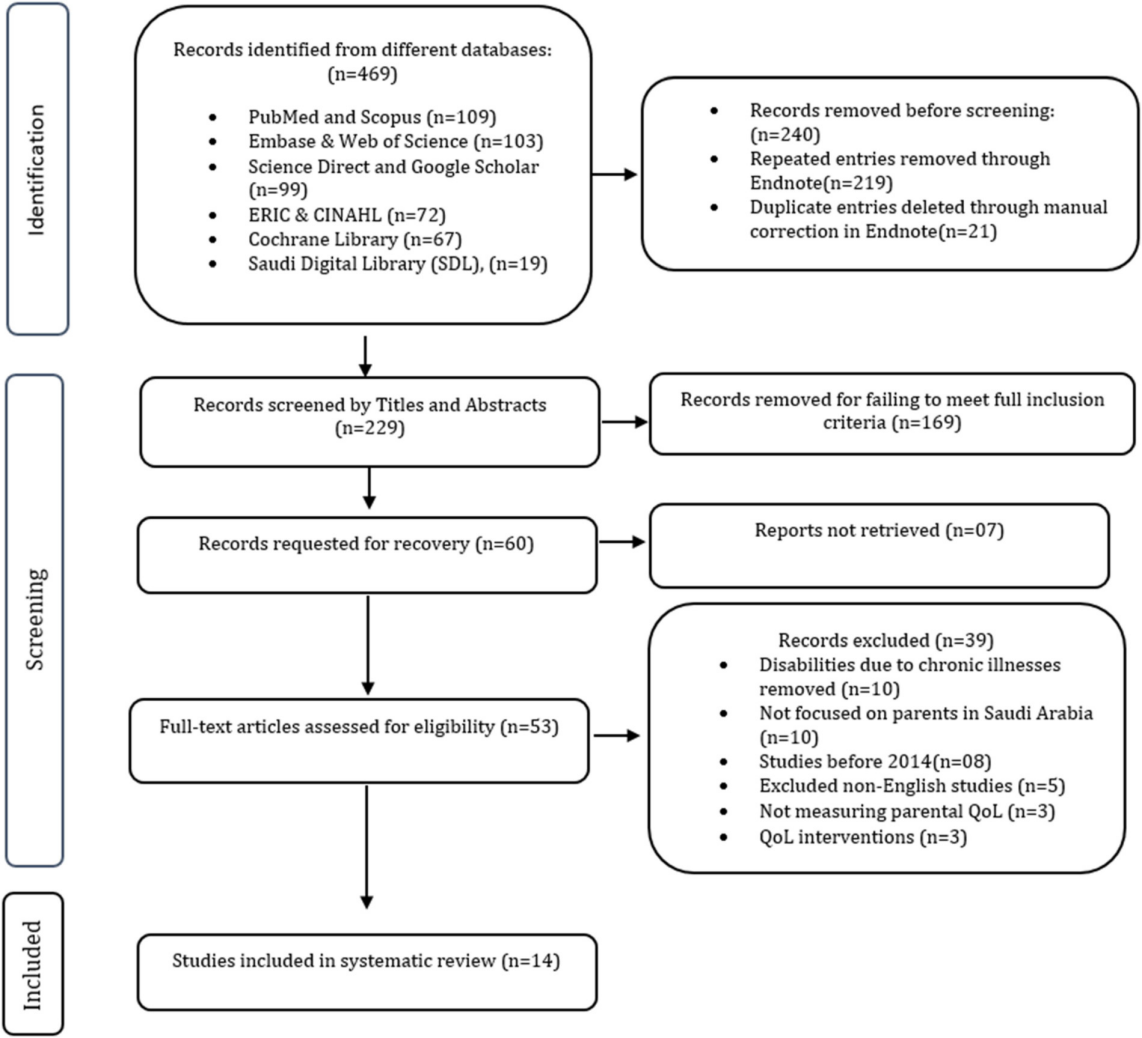
PRISMA diagram.

The Figure 2 illustrates the process of selecting studies for the systematic review. Initially, 469 records were identified from multiple databases. Before screening, 240 duplicate or repeated records were removed, leaving 229 records to be screened by title and abstract, of which 169 were excluded for not meeting the inclusion criteria. Next, 60 full-text articles were requested for review, but 7 could not be retrieved. Out of 53 full-text articles assessed for eligibility, 39 were excluded due to reasons such as disabilities caused by chronic illnesses, studies not focused on parents in Saudi Arabia, studies published before 2014, non-English articles, studies not measuring parental QoL, or those involving QoL interventions. Ultimately, 14 studies were included in the systematic review.

### 4.2 Demographic and clinical characteristics of study participants

The analysis included a total of 1,841 participants, comprising 1,090 mothers (60.18%), 418 fathers (23.08%), and 333 caregivers (18.39%), highlighting the diverse distribution of key demographic groups across the studies (41–54) (see Table 3). The age range for mothers was primarily from their early 20s to over 50 years, with most studies reporting an average age between 30 and 45 years. Similarly, the fathers' ages range from 20 to over 50 years, with most fathers being between 31 and 50 years old. The total number of children with disabilities across these studies is 1460, with common disabilities including ASD, ADHD, CP, and other physical disabilities such as stroke and amputations. The children in these studies generally range from 1 to 18 years old, with the majority falling between 1 and 5 years and 6–10 years. The studies reporting 539 people (46.4%) were employed, while 623 people (53.6%) were unemployed across the studies. Notably, the studies by Zahra et al. (47), Tedla et al. (44), Subke et al. (41), AlAhmari et al. (49), and Alhazmi et al. (54) did not explicitly mention either the employment status of caregivers or support received by families. The QoL measures used across the studies include WHOQOL-BREF, PedsQL 3.0, and RAND SF-36, among others. A variety of support from services was reported, including rehabilitation services, telehealth, special education, and government financial support, although some caregivers faced challenges related to limited access and discrimination. Comorbidities such as learning difficulties, autism, ADHD, and physical impairments were often noted in children with disabilities (see Table 2).

**Table 3:** Summary of studies characteristics of caregivers of children with disabilities: employment Status, comorbidities, and support services.

Ref.	Study DESIGN	Mothers/fathers	Employment (full/part-time)	Type of disability	Age (years)	Age of child with disability (years)	QoL measure	Support from services	**Comorbidities**
Subke et al. (41)	Cross-sectional study	58.5% were mothers, 41.5% were fathers.	56.5% of parents were employed	ASD.	Parents' ages ranged from 23 to 45 years, with a mean age of 34.46 ± 4.7 years.	Children aged between 2 and 14 years, all clinically diagnosed with ASD at least 3 months before the study.	WHOQOL-BREF	N/A	N/A
Alhefdhi et al. (42)	Cross-sectional	Mothers: 66% Fathers: 34%	Teachers: 32%	ADHD	Majority were young to middle-aged adults.	1–5 years: 62%	WHOQOL-BREF (Arabic version)	Limited support from schools; workshops attended by 54%.	ADHD-related psychological burden; discrimination.
			Housewives: 12%			6–10 years: 8%			
			Businesspeople: 8%			11–15 years: 3%			
			Engineers: 3%%			16–18 years: 27%			
			Unemployed: 5						
Tedla et al. (43)	Cross-sectional	Mothers: 111 Fathers: 1 Grandparents/Staff: 20	The study did not explicitly categorize employment as full-time or part-time. However, it mentioned that the majority of caregivers were mothers (111 out of 132 participants)	Cerebral Palsy (CP)	Caregiver age: 41.53 ± 6.78	2–18 years	PedsQL 3.0 CP Module	Rehabilitation and special education services.	N/A
Tedla et al. (44)	Cross-sectional cohort study	All caregivers were women, primarily mothers (85.84%), with 14.16% female maids.	N/A	Cerebral palsy (various types).	Mean caregiver age: 40.38 years (SD ± 7.09).	Mean age of children with CP: 7.55 years (SD ± 3.04), children aged between 2 and 12 years.	WHOQOL-BREF (Arabic version)	55.7% received financial support from the government; 44.3% reported excellent external support.	Not specified
Asdaq et al. (45)	Cross-sectional study	Not specified	34.2% employed; remaining 65.8% were unemployed or inactive.	Physical (35.1%), sensory (24.3%), intellectual, and developmental disabilities.	Majority are over 36 years old.	**N/A**	WHOQOL-BREF	73.9% had access to telehealth services.	75.7% had a concomitant medical condition.
Alasiri et al. (46)	Cross-sectional	Predominantly mothers (59.8%), Fathers accounted for 33.3%, while other relatives (siblings or others) made up a small proportion (6.9%).	Unemployed: 31.4%	ASD	Adults: Majority 30–49 years	Children: 1–14 years	Beach Center Family QoL Scale	Services included speech therapy, behavioral therapy, special education	Severity of ASD noted (Level 1–3); No other comorbidities explicitly listed
			Government sector (full-time/part-time): 40.2%						
			Private sector (full-time/part-time): 17.6%						
			Students: 2.0%						
			Retired: 8.8%						
Zahra et al. (47)	Cross-sectional study	Not specified.	Not specified.	Physical disabilities, including stroke and amputations.	Not specified	Participants aged 15 years and older.	WHOQOL-BREF	Rehabilitation services from Rehabilitation Hospital, Madinah.	Chronic diseases were included in the analysis as influencing PA levels.
Algadeeb et al. (48)	Cross-sectional study	Mothers (46.6%) and fathers (49.5%) comprised the majority of caregivers.	Employed caregivers were the largest group (64.1%), followed by homemakers (26.2%).	ADHD	Caregivers' ages ranged from 14 to 57 years, with a mean age of 36.83 years.	Most children were aged 7–10 years (37.9%), followed by 4–6 years (34.0%).	WHOQOL-BREF	Majority received help from spouses (66%), and some relied on extended family or specialists.	No specific comorbidities were emphasized in the study.
AlAhmari et al. (49)	Cross-sectional	261 caregivers of children with DS	Not specified in detail, general demographic focus on education and income	Down syndrome	Majority above 40 (62.8%); some between 20 and 40 (36%)	0–14 years	WHOQOL-BREF	Limited, with gaps in healthcare access and social integration	Excluded children with additional health issues
Alenezi, S et al. (50)	Cross-sectional study	93 mothers (59.6%) and 63 fathers (40.4%	62.8% employed	ADHD	Mean: 40.9	Mean: 10.03 years	WHOQOL-Brief	Neurodevelopmental centers	Learning difficulties (80.9%), autism (28.2%), separation anxiety (3.6%), Asperger's (3.6%)
Alenazi et al. (51)	Cross-sectional study	Majority were mothers (80.9%), with fathers comprising 19.1%	Most caregivers were unemployed (69.1%), while 30.9% were employed	ASD	Parents' age groups: <40 years (54.8%), ≥40 years (45.2%)	<7 years (32.1%), 7–10 years (42.9%), >10 years (25.0%)	Short-form 36 (SF-36) health survey	Services from the developmental abnormalities clinic and Maternal and Children's Hospital in Arar City	N/A
Sulaimani et al. (52)	Analytical cross-sectional study	Mothers (*n* = 48, 51.6%), Fathers (*n* = 37, 39.8%), Others (*n* = 10, 8.6%)	Employed (*n* = 48, 50.5%), Unemployed (*n* = 47, 49.5%)	Physical disabilities (57.1%), Down syndrome (10.2%), Autism (6.1%)	26–35 years (24.7%), 36–45 years (56.5%), 46–55 years (10.6%), 56+ (8.2%)	1–5 years (15.6%), 6–10 years (40.0%), 11–18 years (44.4%)	RAND SF-36 (Short Form Health Survey)	Services from rehabilitation centers such as KFMC and Alawael Center	Presence of multiple disabilities noted (e.g., children with severe disabilities or comorbid conditions)
Al Awaji et al. (53)	Comparative Cross-Sectional	Mothers only	30.1% of MCD employed (lower than 55.9% in CON)	Autism spectrum disorder (24% of disabilities reported among MCD)	Mothers of Children with Disabilities (MCD): Most ≥36 years	≤18 years	WHOQOL-BREF	Saudi government offers support through rehabilitation centers and annual funds for children with disabilities, though access was limited during a lockdown.	Common comorbidities reported for children with disabilities include Down syndrome (19%), physical impairments (23%), ADHD (7.5%), and sensory impairments (2%).
Alhazmi et al. (54)	a cross-sectional study	51% of fathers and 49% of mothers participated as primary caregivers	Employment status positively impacted the caregivers' quality of life, mainly through financial stability, but specific employment statistics were not provided	Among 54 children diagnosed with ASD by developmental pediatricians, 75.9% were males (41 children) and 24.1% were females (13 children).	The majority of parents in the study were aged 31–40 years (44.4%), followed by 41–50 years (38.9%), with smaller proportions aged 20–30 years (10.2%) and over 50 years (6.5%)	Children aged 1–14 years included in the study	WHOQOL-BREF	The study does not specify government support but suggests that financial stability, possibly through subsidies, improves the caregivers' quality of life	Attention-Deficit/Hyperactivity Disorder (ADHD), Aggression, Defiance, Destructive behaviors, Self-harm behaviors

ASD, Autism spectrum disorder; ADHD, attention-deficit/hyperactivity disorder; CP, cerebral palsy; DS, down syndrome; QoL, quality of life; WHOQOL-BREF, world health organization quality of life–brief version; PedsQL 3.0 CP Module, pediatric quality of life inventory version 3.0 cerebral palsy module; SF-36/RAND SF-36, Short Form Health Survey (36-Item); Beach Center Family QoL Scale, Beach center family quality of life scale; KFMC, king fahad medical city; MCD, mothers of children with disabilities; CON, control group (mothers of children without disabilities); N/A, not available or not reported; SD, standard deviation.

### 4.3 Measurement of QOL

The reviewed studies used different tools to measure QoL across several areas of life. The most common tool was the WHOQOL-BREF, used in 10 studies, which looks at physical, psychological, social, and environmental health. Determinants like employment, education, income, and coping strategies had a big impact on these areas. For instance, Subke et al. (41) and Tedla et al. (43) found that parents who were employed and had higher education reported better QOL, especially in physical and psychological aspects. Another tool, the Beach Center Family QOL Scale, used by Alasiri et al. (46), focused on how the family functions, including family interactions, parenting, and emotional well-being. The PedsQL 3.0 CP Module, applied in Tedla et al. (43), looked specifically at children with cerebral palsy, showing differences in mobility and age-related health outcomes. The SF-36 survey, used by Alenazi et al. (51) and Sulaimani et al. (52), measured physical functioning, role limitations, energy, emotional well-being, and pain, highlighting how caregiving and sociodemographic factors affect parents' lives. Overall, all these tools showed a similar picture: parents' quality of life is strongly influenced by caregiving duties and sociodemographic factors, with physical, psychological, and social areas being the most affected.

### 4.4 Quality appraisal

As part of the quality appraisal, several quality criteria were applied to systematically evaluate each study, and the results are summarized in Table 4.

**Table 4:** Methodological quality assessment and inter-rater agreement of included studies.

Quality rating	Percentage of studies	Studies included	Observations
High (Good)	71.43%	Tedla et al. (43, 44); Subke et al. (41); Zahra et al. (47); Algadeeb et al. (48); Alhazmi et al. (54); Sulaimani et al. (52); Alenezi et al. (50); Alasiri et al. (46); Al Awaji et al. (53)	Studies were rated as high quality based on strong methodology, appropriate sampling strategies, robust data analysis, validated measurement tools, ethical considerations, relevance to QoL, and practical implications. All studies consistently received “Good” ratings in both review rounds (R1 and R2). Control groups were well defined and appropriately matched with disability groups.
Moderate	28.57%	Alhefdhi et al. (42); Asdaq et al. (45); AlAhmari et al. (49); Alenazi et al. (51)	Studies were rated as moderate quality due to some limitations in methodology, sampling procedures, or data analysis. However, all studies clearly stated their research objectives and provided strong rationales for the research.
Inter-rater Reliability	—	—	Two reviewers independently assessed all studies. Inter-rater agreement was strong, with Cohen's Kappa (*κ* = 0.81).

### 4.5 Results from comparative studies

The findings below summarize the domain-specific QoL outcomes across the included studies, highlighting both positive and negative effects identified using different validated assessment instruments among caregivers and children with disabilities in Saudi Arabia (Table 5).

WHOQOL-BREF
  Used in 10 of the 14 studies, making it the most frequently applied QoL instrument.
  Nine studies reported negative outcomes in at least one domain.
  The Physical Health (PHY) domain was most affected, with negative outcomes reported in 5 studies (50%), particularly among mothers.
  Social Health (SOC) and Environmental Health (ENV) domains were negatively affected in 4 studies each (40%).
  The Psychological Health (PSY) domain showed negative outcomes in 3 studies (30%).
SF-36 Health Survey
  Applied in two studies: Sulaimani et al. (52) and Alenazi et al. (51).
  Sulaimani et al. (52) reported positive outcomes in Role Emotional (RE), Mental Health (MH), Vitality/Energy-Fatigue (VT), and Social Functioning (SF).
  In the same study, negative outcomes were observed in Bodily Pain (BP), General Health (GH), and Role Physical (RP), with no change in Physical Functioning (PF).
  Alenazi et al. (51) reported negative outcomes in seven domains, with Bodily Pain (BP) being the only domain showing improvement.
Beach Center Family Quality of Life (BCFQoL) Scale
  Positive outcomes were reported in Family Interaction (FI) and Parenting Practices (PP).
  Negative associations were observed in Emotional Well-Being (EW), Physical/Material Well-Being (PMW), and Disability-Related Support (DRS).
PedsQL 3.0 Cerebral Palsy (CP) Module
  Positive, age-related associations were found in Daily Activities (DA), School Activities (SA), and Speech/Communication (SC).
  Negative outcomes were consistently reported in Mobility and Balance (MB), Pain/Discomfort (PH), Fatigue (FA), and Eating Activities (EA) across age groups.

**Table 5:** Comparison of QOL measures across All included studies on children with disabilities and their caregivers in Saudi Arabia.

				SF								WHOQOL-BREF				BCFQoL Scale					PedsQL 3.0 CP Module						
Ref.	Study Setting/Recruitment	Compared Groups	QoL Measure	VT	SF	RE	MH	PF	RP	BP	GH	PHY	PSY	SOC	ENV	FI	PP	EW	PMW	DRS	DA	SA	MB	PH	FA	EA	SC
(43)	132 CP children aged 2–18 recruited from hospitals and rehabilitation centers in SA.	Compared CP children based on mobility (GMFCS), age (2–18 years), and schooling (normal vs. special).	PedsQL 3.0 CP Module																								
(46)	SA; King Abdulaziz Medical City and online selection	Families of children with ASD	BC Family QoL Scale																								
**(**47)	Disabled from Rehabilitation Hospital; non-disabled online selection in SA	Individuals with physical disabilities (*n* = 116). Individuals without disabilities (*n* = 243).	WHOQOL-BREF									**N/A**	**N/A**	**N/A**													
(45)	Participants recruited from disability centers, hospitals, clinics, charity events, and health camps in SA	Individuals with disabilities, grouped by depression status, vaccination status, and disability severity.	WHOQOL-BREF																								
(44)	Data was collected at hospitals and disability centers over one year in SA.	Caregivers of children with CP, focusing on caregivers' demographics and child-related factors (e.g., mobility, education).	WHOQOL-BREF instrument, Arabic version																								
(42)	100 parents of ADHD children recruited via social media and hospital records in SA	mothers vs. fathers	WHOQOL-BREF (Arabic version)																								
(48)	Conducted at the Developmental and Behavioral Disorders Center, Al-Ahsa; participants recruited via online surveys.	Caregivers of children with ADHD, focusing on sociodemographic variations such as gender, marital status, and employment.	WHOQOL-BREF																								
(49)	Tertiary care hospital in Riyadh; convenience sampling of Down syndrome caregivers.	Not explicitly grouped; all respondents were caregivers of children with Down syndrome	WHOQOL-BREF																								
. (50)	Conducted in SA, cross-sectional design. Recruitment through online surveys sent via neurodevelopmental centers.	Parents of children with ADHD, compared by income level, coping strategies (adaptive vs. maladaptive), gender, and employment status.	WHOQOL-BREF																								
**(**54)	Conducted at King Saud Medical City, Riyadh; recruited parents of children with ASD (1–14 years).	The study analyzed intra-group differences in ASD parents based on socio-demographics.	WHOQOL-BREF																								
(53)	SA, during COVID-19 lockdown. Recruitment via online surveys (WhatsApp, Twitter, Facebook).	MCD vs. CON	WHOQOL-BREF																								
(41)	Conducted in Jeddah, at the Saudi National Center for Developmental and Behavioral Disorders, involving 200 parents of children with ASD.	Compared groups based on demographics: gender (male vs. female), employment (employed vs. unemployed), education level, and housing type.	WHOQOL-BREF																								
(51)	Conducted in Arar City, Saudi Arabia, at Maternal and Children's Hospital (Jan–Mar 2019).	Caregivers with impaired quality of life (63.1%) versus those without impairment.	Short-form 36 (SF-36) health survey with scores below 50 indicating poor QOL.																								
(52)	Rehabilitation centers in SA (Riyadh, Makkah); recruitment via social media and centers.	Mothers vs. other caregivers; Educated vs. non-educated caregivers.	RAND 36-Arabic version).																								

Red areas indicate significantly lower QoL scores and a negative association with the comparative groups, while white areas are not tested. Green areas indicate higher QoL scores and a positive association with the compared groups, blue areas show a moderate association with no positive or negative trend, and brown areas indicate similar results for the comparative groups, showing no statistical significance.

SF, short form health survey (SF-36); WHOQOL-BREF, World health organization quality of life–brief version; BCFQoL, beach center family quality of life scale; PedsQL 3.0 CP Module, pediatric quality of life inventory version 3.0 cerebral palsy module. Domain abbreviations are as follows: VT, vitality/energy–fatigue; SF (domain), social functioning; RE, role emotional; MH, mental health; PF, physical functioning; RP, role physical; BP, bodily pain; GH, general health; PHY, physical health; PSY, psychological health; SOC, social relationships; ENV, environmental health; FI, family interaction; PP, parenting practices; EW, emotional well-being; PMW, physical/material well-being; DRS, disability-related support; DA, daily activities; SA, school activities; MB, mobility and balance; PH, pain/discomfort; FA, fatigue; EA, eating activities; SC, speech/communication.

## 5 Findings

### 5.1 Questions 1 and 2: results from the comparative studies

How does caregiver QOL vary by sociodemographic factors (e.g., gender, employment, education, marital status, income)?
How do different disability types and severity levels affect caregiver burden and QOL?

A systematic review was conducted to summarize findings on the QoL of caregivers of children with disabilities in Saudi Arabia. It has been observed that caregiver QOL is influenced by sociodemographic factors, such as gender, employment, education, marital status, and income, as well as by the type and severity of the child's disability (see Table 5). For example:

Alhefdhi et al. (42) found lower QOL among single and unemployed mothers. Marital status, occupation, and number of children were significant predictors. The environmental domain was most negatively affected, particularly in mothers facing economic or social challenges (*p* < 0.05).
Sulaimani et al. (52) reported that educated caregivers had higher emotional well-being (53.57 vs. 33.33; *p* = 0.027). Caregivers of children with severe disabilities had lower bodily pain scores (58.33 vs. 80.00; *p* = 0.047). General health was also lower in those caring for more than one disabled child.
Subke et al. (41) showed that employed parents had higher QOL in physical (*p* = 0.002), psychological (*p* = 0.008), and social domains (*p* < 0.001). Higher education and homeownership were associated with better psychological well-being (*p* < 0.001 and *p* = 0.035, respectively). Age of parent or child had no significant impact.
Tedla et al. (44) assessed 106 female caregivers of children with CP, with an average QOL score of 66.38/100. Physical health was most affected (62.45), followed by psychological (65.44), social (72.83), and environmental domains (64.69). Significant predictors of higher QOL included child mobility (*p* < 0.001), caregiver education (*p* < 0.001), and school attendance. Socioeconomic factors, like income and housing, also contributed.
Algadeeb et al. (48) examined caregivers of children with ADHD and reported that all QOL domains were negatively impacted, especially environmental (mean = 60.68). Most caregivers experienced moderate to high burden (86.4%), with divorced caregivers facing the highest stress (*p* = 0.024). Psychological and social QOL varied significantly by marital status and age (*p* = 0.012 and *p* = 0.024). Family dysfunction was common (87.4%).

### 5.2 Question 3: are there differences in QoL outcomes for mothers compared to other caregivers, such as fathers or extended family members?

This section examines whether QoL outcomes differ between mothers and other caregivers, including fathers and extended family members, based on evidence from the included comparative studies. Overall, most studies indicate that mothers experience poorer QoL outcomes than other caregivers.

Ten studies using the WHOQOL-BREF reported consistent differences in QoL outcomes between mothers and other caregivers, with mothers generally reporting lower scores across multiple domains.
**For example**, Subke et al. (41) conducted a cross-sectional study in Jeddah involving 200 parents of children with (ASD), where mothers accounted for 58.5% of participants and were mainly aged between 23 and 45 years. The study found that male parents reported significantly better social relationship scores than female parents, indicating lower social QoL among mothers (*p* = 0.002).
**Similarly**, Algadeeb et al. (48) reported that mothers of children with ADHD consistently scored lower than fathers across all WHOQOL-BREF domains. Mothers had lower physical health scores (57.51 ± 20.14 vs. 62.32 ± 20.71), psychological health scores (57.12 ± 16.24 vs. 61.60 ± 13.13), and social relationship scores (57.64 ± 24.06 vs. 63.89 ± 22.83), with the largest difference observed in the social domain.
**Additionally**, Alenezi et al. (50) confirmed these gender-based disparities by showing that mothers had significantly lower overall QoL scores than fathers (49.3 ± 11.8 vs. 55.6 ± 12.2; *p* = 0.031). The psychological health domain showed the greatest disparity, with mothers scoring 44.7 ± 10.5 compared to 50.3 ± 11.2 for fathers (*p* = 0.024), and this domain was strongly associated with overall QoL (r = 0.859, *p* < 0.01).
**In contrast**, Alhefdhi et al. (42) reported no statistically significant differences in QoL between mothers and fathers (*p* = 0.833). Although mothers showed slightly higher physical and psychological scores and fathers had marginally higher environmental scores, these differences did not reach statistical significance.
**Furthermore**, using the SF-36 questionnaire, Sulaimani et al. (52) observed notable emotional well-being differences among caregivers. Mothers scored lower (mean ± SD: 60.33 ± 20.31) compared to siblings such as brothers (mean ± SD: 74.00 ± 8.49; *p* = 0.023), highlighting the heightened emotional burden experienced by mothers as primary caregivers.

These findings are consistent with broader literature indicating that mothers, as the primary caregivers, often face greater emotional and caregiving burdens than other family members. Their responsibilities typically encompass daily care management, coordinating medical appointments, and providing emotional support, all while balancing household and occupational roles. The cumulative effect of these duties likely contributes to the consistently lower emotional well-being scores observed among mothers (55–59).

### 5.3 Research 4: which quality of life domains (e.g., physical, psychological, social, environmental) are affected most?

To answer the fourth research question, the results show that QoL affects caregivers in different ways across several areas. Physical health was one of the most challenging areas, especially for caregivers looking after children with severe disabilities or limited mobility. Many caregivers reported feeling physically exhausted and experiencing health problems due to the constant demands of caregiving [Tedla et al. (43), Sulaimani et al. 52]. Psychological well-being was also strongly affected, with caregivers reporting high levels of stress, anxiety, and depression. Mothers and caregivers who relied on poor coping strategies were particularly vulnerable and reported lower psychological QoL [Alenezi et al. (50), Subke et al. 41]. Social relationships were another area of difficulty, as many caregivers felt isolated and disconnected from others. However, those who took part in support groups reported better social QoL because these groups helped them feel more connected and less alone [Alhazmi et al. (54), Al Awaji et al. 53]. Environmental QoL was closely linked to practical factors such as housing, safety, and access to services. Caregivers with better financial stability and access to resources tended to report better outcomes in this area (Alhazmi et al. (54), Zahra et al. (47). Overall, these findings show that caregivers face different challenges across multiple areas of quality of life, rather than in just one single domain (see Table 5).

### 5.4 Research question 5: how do social and governmental support systems (such as rehabilitation services and financial aid) affect the QoL of parents of children with disabilities in Saudi Arabia?

Social and governmental support systems, such as rehabilitation services and financial aid, significantly enhance the QoL for parents of children with disabilities in Saudi Arabia. Parents who received access to rehabilitation services reported better QoL. For example, Tedla et al. (43) noted that caregivers of children with CP benefited from rehabilitation and special education services, which contributed positively to their QoL. Financial support was shown to significantly alleviate stress. In Tedla et al. (44), 55.7% of caregivers received government financial support, which correlated with improved QoL measures. Alhazmi et al. (54) also highlighted the role of financial stability, often enabled by employment or subsidies, in improving caregivers' QoL across multiple domains. Although the Saudi government provides funds and access to rehabilitation centers [Al Awaji et al. (53)], gaps remain in the accessibility and availability of services, particularly during lockdowns or crises, reducing the potential benefits for caregivers. AlAhmari et al. (49) emphasized limited healthcare access and social integration gaps, negatively impacting caregivers' QoL. Speech and behavioral therapy, as noted by Alasiri et al. (46), were crucial for parents of children with autism, leading to enhanced family satisfaction.

## 6 Discussion

It is noteworthy that, in this systematic review, the QoL of parents and caregivers of children with disabilities in Saudi Arabia has been shown to be influenced by a combination of sociodemographic, contextual, and structural factors (14, 23, 24, 26, 31, 41, 52). There has been a growing interest in caregiver well-being, particularly in relation to long-term caregiving responsibilities; therefore, the available evidence was reviewed and summarized in order to understand how these factors are associated with caregiver QoL and to what extent such associations are evident. Across the included studies, higher levels of employment, income, and educational attainment were found to be consistently associated with better QoL outcomes (24, 26, 37, 48, 49, 54). In addition, differences across physical, psychological, social, and environmental QoL domains were observed to be associated with caregiver gender and marital status. These patterns have also been reported in international literature, in which socioeconomic position has been identified as a key determinant of caregiver well-being and the unequal distribution of caregiving burden across populations has been highlighted (10, 13, 27–29).

The reduction in parental QoL that has been reported is associated with the continuous and demanding role of caring for a child with long-term health or developmental conditions (26, 28, 29, 41, 52). Emotional, physical, and financial strain is often experienced by caregivers, which can build up over time and thereby lead to chronic stress. Studies have shown that mothers, who are more often the primary caregivers, are particularly vulnerable and more likely to report higher psychological distress (14, 23, 26, 31, 53). This is particularly significant, as increased anxiety, depression, and parent–child strain has been recorded in families of children with special needs in comparison with families of typically developing children (55, 56). Despite these challenges, caregiving experiences are not only variable but also affected by the availability of social support and access to services, which can be used to reduce negative outcomes. However, social stigma and discrimination have been reported among families of children with intellectual disabilities or autism, with cultural norms shaping how stress is experienced and expressed, thereby further impacting QoL outcomes (57, 58). In the context of these findings, it becomes clear that caregiving stress is caused not only by the child's condition but also by social and environmental factors, which must be addressed for better parental well-being. More recently, it has been suggested that programs offering social, emotional, and educational support could thus help improve parental QoL (10, 14, 28, 52).

Related to that, international evidence has provided important comparative perspectives, which can be used to contextualize the Saudi findings (10, 27–30). This body of research has examined caregiver QoL across different sociocultural and economic settings and has assessed how it is shaped in response to variations in support systems (10, 13, 27–29). For example, in the study by Xia et al. (59), which examined caregivers of children with disabilities in China, anxiety and depression were reported in 33.6% and 36.1% of caregivers, respectively. It was shown that mental health-related QoL was improved through psychological and financial support; however, physical QoL was not significantly affected. Rehabilitation services and financial assistance were identified as the most needed forms of support, particularly among parents and grandparents. Similarly, in the study by Scheibner et al. (60), parenting stress among German caregivers was analysed, and significantly higher stress levels were reported among mothers and single parents compared with fathers and two-parent families. Stress was shown to be highest during children's early school years and was associated with employment type, as part-time employment was linked to higher stress levels compared with unemployment or full-time employment. In the Malaysian context, QoL among caregivers was assessed by Ismail et al. (61), and it was found that low-income families and those caring for children with comorbidities or feeding difficulties were the most adversely affected. While psychological well-being (68.98) and social relationships (75.15) were reported to score relatively higher, environmental support (64.61) was more limited, indicating that financial strain and restricted resources contributed to poorer QoL. Taken together, these studies have demonstrated that targeted and context-specific support systems are essential for reducing caregiver burden and improving QoL outcomes (61).

Child-related factors have also been shown to play an important role in affecting caregiver QoL, especially depending on the type and severity of the child's disability. This association has been supported by several previous studies (6, 15, 16, 21, 22, 25, 31, 43, 44, 46). In a review by Isa et al. (62), which looked at caregivers of children with ASD, ADHD, and CP, it was shown that ASD is associated with very high caregiving demands. Children with ASD were reported to need constant supervision and special support, thereby increasing emotional strain and the effort of caregiving. As a result, sleep problems, financial pressure, and social isolation were often reported by caregivers. These challenges were further increased by the need to manage behavioural and social difficulties related to ASD, making daily caregiving especially demanding. Caring for children with ADHD was also shown to lower caregiver QoL, as hyperactivity, impulsivity, and attention difficulties must be managed continuously at home and school, which can cause ongoing stress, tiredness, and family conflicts (63, 64). In addition, children with physical disabilities were found to depend heavily on caregivers for moving around, personal care, and medical support, which can be physically tiring. The extra time and cost for therapy sessions, medications, special education, and assistive devices were shown to further increase caregiver strain and lower overall QoL (62). This is particularly significant, as these challenges must be addressed to improve caregiver well-being. In the context of these findings, it becomes clear that child-related characteristics are not only a key cause of caregiver stress but also show areas where support programs could be used to improve QoL (62).

These findings matter for Saudi Vision 2030, which focuses on improving health and social care (1, 2, 5, 7, 8). While most programs target children, supporting caregivers is essential for better care outcomes (14, 23, 31, 44, 52). As noted by Alharbi et al. (65), Vision 2030 includes grants, subsidies, universal healthcare, and education reforms, which are designed to reduce psychological, economic, and social burdens on families. However, gaps in service availability and poor coordination across health, education, and social sectors must be addressed to improve caregiver QoL fairly across the country. Following this, practical strategies could be used, such as adding caregiver well-being checks in paediatric and rehabilitation services, expanding community rehabilitation programs, improving financial support, and increasing peer-support as well as respite care. In this context, it becomes clear that supporting caregivers is particularly significant, enabling better outcomes for both children and families and helping achieve the goals of Vision 2030 (65).

### 6.1 Strengths and limitations

The strengths of this study include its systematic and comprehensive approach, following PRISMA guidelines to synthesize evidence from multiple Saudi-based studies published between 2020 and 2024, which enhances methodological rigor and transparency (40). The inclusion of diverse childhood disabilities and the use of validated QoL instruments allow for meaningful comparisons across different caregiver groups and life domains. Additionally, by focusing specifically on the Saudi context, this review fills an important research gap and provides culturally relevant insights (1, 3, 65), while identifying key vulnerable groups and highlighting the role of support services, making the findings highly relevant for healthcare policy, family support planning, and future research (8, 14, 52).

Despite these strengths, several limitations must be addressed for proper interpretation of the findings. Most importantly, cross-sectional designs were employed in all included studies, which restricts causal relationships between caregiving factors and parental QoL from being inferred (16, 27, 62). It is straightforward that assessment of how caregiver QoL evolves over time or in response to changes in disability severity, service access, or policy interventions is prevented by the lack of longitudinal evidence, which must be addressed in future research (30, 55). When these changes are considered, it becomes evident that causal links cannot be established.

QoL outcomes were predominantly based on self-reported measures, which can be influenced by recall bias, response bias, and social desirability effects (55, 56). Cultural norms within Saudi society may further affect how psychological distress or social challenges are reported by caregivers, which may have led to under- or overestimation of certain QoL domains. This is particularly significant, as uncertainty in the reported outcomes could thereby be increased (59).

A clear gender imbalance was observed, with mothers comprising the majority of participants, which is one challenge in the included studies. While real-world caregiving patterns are reflected, generalizable findings for fathers and other caregivers are not always obtained, thereby restricting deeper gender-comparative analyses (31, 60). Following this, it becomes apparent that gender differences in caregiver QoL should be explored in future research in order to understand the full scope of caregiving experiences and to what extent these experiences vary by caregiver type (23, 28).

Methodological heterogeneity, which included differences in QoL instruments, reporting formats, and domain-level analyses, limited direct comparability and precluded quantitative meta-analysis (27, 30, 40). Convenience sampling from single regions or clinical settings was used in several studies, which reduced national representativeness and limited insights into rural or underserved populations. It was shown that observed patterns of caregiver QoL could thereby be influenced, which must be considered when interpreting the results. Finally, some studies lacked detailed diagnoses or comorbidity data, which may have affected caregiver QoL and should be addressed in future research (16, 43, 44).

### 6.2 Challenges and future directions

The QoL of parents caring for children with disabilities in Saudi Arabia is affected by several ongoing challenges. Services are not evenly distributed, with families in urban areas having better access than those in rural regions, especially for specialized therapies such as speech and language services (23, 66). Financial support often does not fully cover the high cost of care and long-term therapy, which places added stress on families. There is also a shortage of trained professionals and specialized centers, making it harder for families to receive consistent and high-quality support (65, 66). Cultural attitudes toward disability can further increase emotional strain and limit social participation for caregivers. Looking ahead, future efforts should focus on improving access to services across all regions, expanding training programs to build a skilled workforce, and strengthening community-based rehabilitation services to better support families and improve caregiver QoL.

## 7 Conclusion

This review shows that taking care of a child with a disability lowers the QoL of parents and caregivers in Saudi Arabia. It was found that physical, mental, social, and environmental well-being are all affected. Mothers are the most affected because they are the main caregivers and often have lower scores than fathers. More than half of caregivers do not have a job, and poor quality of life is linked to unemployment, low income, less education, and little support. Caring for children with severe or multiple disabilities increases stress and burden. Physical health is the most affected, followed by social and environmental life, and mental stress is common. Some support services exist, but they are not always enough or easy to access. This shows that social and financial problems make life harder for caregivers. It is important to provide more financial help, better access to rehabilitation and mental health services, and special support for mothers and disadvantaged families to improve caregivers' quality of life in Saudi Arabia.

## Data Availability

The raw data supporting the conclusions of this article will be made available by the authors, without undue reservation.

## References

[1] Malik S Shrahili M Khan SU. The transformation to reduce the disability prevalence rates in Saudi Arabia: a theoretical analysis. J Disabil Res. (2024) 3(3):20240024. 10.57197/JDR-2024-0024

[2] Yousef L Almatroodi N AlAngari D AlShehri R Alshammari S. Disability inclusion assessment in primary healthcare centers in eastern Saudi Arabia: a way forward. J Med Life. (2023) 16(12):1813. 10.25122/jml-2023-0385 38585520 PMC10994619

[3] Alghadier M Albesher RA Basoudan R. Bibliometric analysis of scientific research on children’s disability in Saudi Arabia. J Disabil Res. (2024) 3(6):20240068. 10.57197/JDR-2024-0068

[4] Almalki NS Arrushaid OM Farah Bakhiet S Alkathiri S. Examining the current practices of the individualized family services plan with young children with disabilities in Saudi Arabia. Int J Dev Disabil. (2023) 69(2):163–78. 10.1080/20473869.2021.1936849 37025331 PMC10071896

[5] General Authority for Statistics, Kingdom of Saudi Arabia. General Authority for Statistics (2025). Available online at: https://www.stats.gov.sa/ (Accessed January 15, 2025).

[6] Alarifi S Denne L Hastings RP. Challenging behaviour and its correlates in preschool-aged children with an intellectual disability in Saudi Arabia. J Intellect Disabil Res. (2024) 68(3):264–76. 10.1111/jir.13109 38052235

[7] Abed MG Abed LG Shackelford TK. A qualitative, small-sample study of employment challenges for people with disabilities in Saudi Arabia. Healthcare (Basel). (2024) 12(3):346. 38338230 10.3390/healthcare12030346 PMC10855392

[8] Medabesh AM Malik NN Shafi M Rashid J. Strategies for facilitating social integration of children with disabilities in Saudi Arabia. J Disabil Res. (2024) 3(7):20240089. 10.57197/JDR-2024-0089

[9] Alghamdi S Alsaigh R. Determinants of physical activity among children with disabilities. Healthcare (Basel). (2023) 11(4):494. 36833029 10.3390/healthcare11040494 PMC9956278

[10] Tétreault S Blais-Michaud S Marier Deschênes P Beaupré P Gascon H Boucher N How to support families of children with disabilities? An exploratory study of social support services. Child Fam Soc Work. (2014) 19(3):272–81. 10.1111/j.1365-2206.2012.00898.x

[11] Alsrhane A Alqudah D. The quality of the application of Saudi building code for children withdisabilities in Saudi Arabia; obstacles, challenges and proposed solutions: parents experiences. Al-Balqa J Res Studs. (2024) 27(1):235–55. 10.35875/1105.027.001.014

[12] Poch AL Alzahrani D Aljuwayhir J Alnahari M. Educating students with learning difficulties in the kingdom of Saudi Arabia. Interv Sch Clin. (2023) 58(4):287–92. 10.1177/10534512221093779

[13] Boelsma F Caubo-Damen I Schippers A Dane M Abma TA. Rethinking FQoL: the dynamic interplay between individual and family quality of life. J Policy Pract Intellect Disabil. (2017) 14(1):31–8. 10.1111/jppi.12224

[14] Alnahdi G. Enhancing the quality of life of mothers of children with intellectual disabilities or autism: the role of disability-specific support. Res Dev Disabil. (2024) 151:104780. 10.1016/j.ridd.2024.104780 38870674

[15] McDougal E Tai C Stewart TM Booth JN Rhodes SM. Understanding and supporting attention deficit hyperactivity disorder (ADHD) in the primary school classroom: perspectives of children with ADHD and their teachers. J Autism Dev Disord. (2023) 53(9):3406–21. 10.1007/s10803-022-05639-3 35776263 PMC10465390

[16] Ostojic K Karem I Paget SP Berg A Dee-Price BJ Lingam R Social determinants of health for children with cerebral palsy and their families. Dev Med Child Neurol. (2024) 66(1):32–40. 10.1111/dmcn.15640 37179527

[17] Centre for Reviews and Dissemination, University of York. PROSPERO International prospective register of systematic reviews (2025). Available online at: https://www.crd.york.ac.uk/PROSPERO/search (Accessed January 20, 2025).

[18] Ewald H Klerings I Wagner G Heise TL Stratil JM Lhachimi SK Searching two or more databases decreased the risk of missing relevant studies: a metaresearch study. J Clin Epidemiol. (2022) 149:154–64. 10.1016/j.jclinepi.2022.05.022 35654269

[19] Tran L Tam DNH Elshafay A Dang T Hirayama K Huy NT. Quality assessment tools used in systematic reviews of *in vitro* studies: a systematic review. BMC Med Res Methodol. (2021) 21(1):101. 10.1186/s12874-021-01295-w 33964880 PMC8106836

[20] Alhuzimi T. Stress and emotional wellbeing of parents due to change in routine for children with autism Spectrum disorder (ASD) at home during COVID-19 pandemic in Saudi Arabia. Res Dev Disabil. (2021) 108:103822. 10.1016/j.ridd.2020.103822 33271447

[21] AlBatti TH Alsaghan LB Alsharif MF Alharbi JS BinOmair AI Alghurair HA Prevalence of autism spectrum disorder among Saudi children between 2 and 4 years old in Riyadh. Asian J Psychiatr. (2022) 71:103054. 10.1016/j.ajp.2022.103054 35248844

[22] Waizbard-Bartov E Fein D Lord C Amaral DG. Autism severity and its relationship to disability. Autism Res. (2023) 16(4):685–96. 10.1002/aur.2898 36786314 PMC10500663

[23] Alnahdi GH Alwadei A Schwab S. Family quality of life of caregivers of individuals with autism, with other disabilities, and without disabilities: the case of Saudi Arabia. Int J Dev Disabil. (2024) 70(6):1010–21. 10.1080/20473869.2024.2362007 39564195 PMC11571730

[24] Al-Janabi RAA Saleem ZA Lami F Jaber OA Jasim SM Dehghan Nayeri N Demographic, ecological and social predictors of quality of life among parents of autistic children: a multi-centre cross-sectional study. Autism. (2025) 29:2058–71. 10.1177/13623613251334166 40346795

[25] Peñuelas-Calvo I Palomar-Ciria N Porras-Segovia A Miguélez-Fernández C Baltasar-Tello I Perez-Colmenero S Impact of ADHD symptoms on family functioning, family burden and parents’ quality of life in a hospital area in Spain. Eur J Psychiatry. (2021) 35(3):166–72. 10.1016/j.ejpsy.2020.10.003

[26] Alsaad A Alramadhan N Aljumah M Alghareeb F Alghadeer H. The effect of the parents’ anxiety and depression on the quality of life of children with cerebral palsy in Al Ahsa, Saudi Arabia. Med Sci. (2021) 25:2778–88.

[27] Garcia Rodrigues M Monteiro Soares M Rodrigues JD Azevedo LF Pereira Rodrigues P Areias JC Quality of life of parents with children with congenital abnormalities: a systematic review with meta-analysis of assessment methods and levels of quality of life. Qual Life Res. (2022) 31(4):991–1011. 10.1007/s11136-021-02986-z. 34482484

[28] Emam MM Al-Hendawi M Gaafar Ali D. Parenting stress and life satisfaction in families of children with disabilities: the mediating effect of social support in three Arab speaking countries. J Fam Stud. (2023) 29(1):134–52. 10.1080/13229400.2021.1893791

[29] Manee F Ateya Y Rassafiani M. A comparison of the quality of life of Arab mothers of children with and without chronic disabilities. Phys Occup Ther Pediatr. (2016) 36(3):260–71. 10.3109/01942638.2015.1076558 26566139

[30] Dalky HF Meininger JC Al-Ali NM. The reliability and validity of the arabic world health organization quality of life-BREF instrument among family caregivers of relatives with psychiatric illnesses in Jordan. J Nurs Res. (2017) 25(3):224–30. 10.1097/JNR.0000000000000146 28481818

[31] Romaniuk A Ward M Henrikson B Cochrane K Theule J. Family quality of life perceived by mothers of children with ASD and ADHD. Child Psychiatry Hum Dev. (2024) 55(2):510–9. 10.1007/s10578-022-01422-8 36074211 PMC9452860

[32] Alhammad SA Alqahtani AS Alwadeai KS Algabbani MF Alhusaini AA. Walking capacity and its association with quality of life among children with down syndrome in Saudi Arabia. BMC Pediatr. (2024) 24(1):55. 10.1186/s12887-023-04519-8 38238665 PMC10797893

[33] Ashina M Katsarava Z Do TP Buse DC Pozo-Rosich P Özge A Migraine: epidemiology and systems of care. Lancet. (2021) 397(10283):1485–95. 10.1016/S0140-6736(20)32160-7 33773613

[34] Steiner TJ Stovner LJ. Global epidemiology of migraine and its implications for public health and health policy. Nat Rev Neurol. (2023) 19(2):109–17. 10.1038/s41582-022-00763-1 36693999

[35] Lombard L Farrar M Ye W Kim Y Cotton S Buchanan AS A global real-world assessment of the impact on health-related quality of life and work productivity of migraine in patients with insufficient versus good response to triptan medication. J Headache Pain. (2020) 21:1–16. 10.1186/s10194-020-01110-9 32349662 PMC7189443

[36] Al Ghadeer HA AlSalman SA Albaqshi FM Alsuliman SR Alsowailem FA Albusror HA Quality of life and disability among migraine patients: a single-center study in AlAhsa, Saudi Arabia. Cureus. (2021) 13(11):e19210. 10.7759/cureus.19210 34873540 PMC8638781

[37] Asiri F Tedla JS Sangadala DR Reddy RS Alshahrani MS Gular K Quality of life among caregivers of children with disabilities in the kingdom of Saudi Arabia: a systematic review. J Disabil Res. (2023) 2(2):8–17. 10.57197/JDR-2023-0016

[38] Alqahtani AS Algabbani MF Alhammad SA Alwadeai KS Alhusaini A. Physical activity status and its association with quality of life among children with down syndrome in Saudi Arabia: a comparative cross-sectional study. PLoS One. (2024) 19(2):e0297111. 10.1371/journal.pone.0297111 38346033 PMC10861077

[39] Alghamdi SA Assiri MI Fallatah RA Albeladi FM Alabdulaziz H Alsaggaf F. Health-promoting activities among Saudi Arabian parents of children with disabilities: a cross-sectional study. Belitung Nurs J. (2025) 11(1):75. 10.33546/bnj.3624 39877218 PMC11770259

[40] Page MJ McKenzie JE Bossuyt PM Boutron I Hoffmann TC Mulrow CD The PRISMA 2020 statement: an updated guideline for reporting systematic reviews. Br Med J. (2021) 372:n71. 10.1136/bmj.n71 33782057 PMC8005924

[41] Subke AA Khan AA Alharthi SA. Factors affecting quality of life among parents having a child with autism spectrum disorder in Jeddah, 2019 (cross-sectional study). J Prev Med Holist Health. (2020) 6(1):27–36. 10.18231/j.jpmhh.2020.006

[42] Alhefdhi H Alshehri N Al Zomia A Lahiq L Hussain A Alaskari A Exploring quality of life, discrimination, and knowledge of parents of ADHD children in Saudi Arabia: a cross-sectional study. Medicine (Baltimore). (2024) 103(24):e38102. 10.1097/MD.0000000000038102 38875372 PMC11175895

[43] Tedla JS Sangadala DR Asiri F Alshahrani MS Alkhamis BA Reddy RS Quality of life among children with cerebral palsy in the kingdom of Saudi Arabia and Various factors influencing it: a cross-sectional study. J Disabil Res. (2024) 3(4):20240050. 10.57197/JDR-2024-0050

[44] Tedla JS Asiri F Reddy RS Gular K Kakaraparthi VN Sangadala DR Caregiver’s quality of life among children with cerebral palsy in the kingdom of Saudi Arabia, and Various influencing factors: a single cohort study. J Multidiscip Healthc. (2023) 16:3705–14. 10.2147/JMDH.S440190 38050486 PMC10693754

[45] Asdaq SMB Alshehri S Alajlan SA Hasino FH Alhammad SK Al Anazi TS Deciphering the correlation of depression, quality of life, and COVID-19 challenges in people with disability: a Saudi Arabian perspective. J Infect Public Health. (2024) 17(6):1013–22. 10.1016/j.jiph.2024.04.008 38636312

[46] Alasiri RM Albarrak DA Alghaith DM Alsayari OS Alqahtani YS Bafarat AY Quality of life of autistic children and supported programs in Saudi Arabia: a cross-sectional study. Cureus. (2024) 16(1):e51645. 10.7759/cureus.51645 38313888 PMC10837822

[47] Zahra A Hassan MS Park JH Hassan SUN Parveen N. Role of environmental quality of life in physical activity status of individuals with and without physical disabilities in Saudi Arabia. Int J Environ Res Public Health. (2022) 19(7):4228. 10.3390/ijerph19074228 35409909 PMC8998774

[48] Algadeeb J AlSaleh EM AlGadeeb RB Alkhoufi HAS Alsaad AJ. Assessment of the quality of life and family function in attention deficit hyperactivity disorder caregivers in Al-Ahsa, Saudi Arabia. Cureus. (2024) 16(9):e70161. 10.7759/cureus.70161 39463622 PMC11504951

[49] AlAhmari FS Alageel AF Aldosari MA Bagha MY. The quality of life of parents of children with down syndrome in a tertiary care hospital: a qualitative research study at Saudi Arabia. Ann Med Surg. (2022) 81:104428. 10.1016/j.amsu.2022.104428 PMC9486666 36147136

[50] Alenezi S Alkhawashki SH Alkhorayef M Alarifi S Alsahil S Alhaqbani R The ripple effect: quality of life and mental health of parents of children with attention deficit hyperactivity disorder in Saudi Arabia: a cross-sectional study. Children. (2024) 11(6):678. 10.3390/children11060678 38929257 PMC11201602

[51] Alenazi DS Hammad SM Mohamed AE. Effect of autism on parental quality of life in Arar city, Saudi Arabia. J Family Community Med. (2020) 27(1):15–22. 10.4103/jfcm.JFCM_157_19 32030074 PMC6984034

[52] Sulaimani GH Kamel S Alotaibi G Telmesani N. Quality of life among family caregivers of disabled children in Saudi Arabia. Cureus. (2023) 15(7):e41320. 10.7759/cureus.41320 37539394 PMC10395756

[53] Al Awaji N Aldhahi M Akil S Awad S Mortada E. Quality of life, needs and fears of mothers of children with disabilities in Saudi Arabia during the COVID-19 lockdown. Int J Environ Res Public Health. (2021) 18(21):11442. 10.3390/ijerph182111442 34769958 PMC8583177

[54] Alhazmi A Alduraibi A Alhemaid M Albreakan A Alshaqha R. Quality of life among the parents of Saudi Arabian children with autistic spectrum disorder, Riyadh, KSA. Middle East J Fam Med. (2023) 21:101–15. 10.5742/MEWFM.2023.95256038

[55] Feizi A Najmi B Salesi A Chorami M Hoveidafar R. Parenting stress among mothers of children with different physical, mental, and psychological problems. J Res Med Sci. (2014) 19(2):145. 24778669 PMC3999601

[56] Hsiao YJ. Parental stress in families of children with disabilities. Interv Sch Clin. (2018) 53(4):201–5. 10.1177/1053451217712956

[57] Mitter N Ali A Scior K. Stigma experienced by families of individuals with intellectual disabilities and autism: a systematic review. Res Dev Disabil. (2019) 89:10–21. 10.1016/j.ridd.2019.03.001 30875608

[58] Smythe T Adelson JD Polack S. Systematic review of interventions for reducing stigma experienced by children with disabilities and their families in low-and middle-income countries: state of the evidence. Trop Med Int Health. (2020) 25(5):508–24. 10.1111/tmi.13388 32145136

[59] Xia C Wei T Tang Q Zheng H Sun M Chen G Anxiety, depression, quality of life, and family support among family caregivers of children with disabilities. Int J Gen Med. (2023) 16:5063–75. 10.2147/IJGM.S434900 37942475 PMC10629400

[60] Scheibner M Scheibner C Hornemann F Arélin M Hennig YD Kiep H The impact of demographic characteristics on parenting stress among parents of children with disabilities: a cross-sectional study. Children. (2024) 11(2):239. 10.3390/children11020239 38397351 PMC10887938

[61] Ismail KF Martine SE Ahmad A Harith S Lua PL Ibrahim WPW. Association of socio-demographic, clinical and feeding characteristics with quality of life among caregivers of disabled children in terengganu, Malaysia. Mal J Public Health Med. (2023) 23(1):207–17. 10.37268/mjphm/vol.23/no.1/art.1009

[62] Isa SNI Ishak I Ab Rahman A Saat NZM Din NC Lubis SH Health and quality of life among the caregivers of children with disabilities: a review of literature. Asian J Psychiatr. (2016) 23:71–7. 10.1016/j.ajp.2016.07.007 27969083

[63] Asa GA Fauk NK Ward PR Mwanri L. The psychosocial and economic impacts on female caregivers and families caring for children with a disability in Belu district, Indonesia. PLoS One. (2020) 15(11):e0240921. 10.1371/journal.pone.0240921 33147246 PMC7641436

[64] Daniel JA Okefienam EC Ugorji TN Agbasi PU Onyido DC Odoh IO Challenges faced by Nigerian parents with disabled children in caring for them. Open J Soc Sci. (2021) 9(10):201–12. 10.4236/jss.2021.910015

[65] Alharbi BA Ibrahem UM Diab HM. Utilizing semantic web for improving quality of life among family caregivers of disabled children: a review in context of Saudi vision 2030. J Disabil Res. (2024) 3(5):20240059. 10.57197/JDR-2024-0059

[66] Al Awaji NN Al-Taleb SM Albagawi TO Alshammari MT Sharar FA Mortada EM. Evaluating Parents’ concerns, needs, and levels of satisfaction with the services provided for ASD children in Saudi Arabia. J Multidiscip Healthc. (2024) 17:123–46. 10.2147/JMDH.S447151 38222477 PMC10785689

